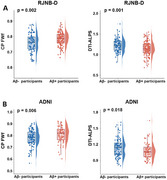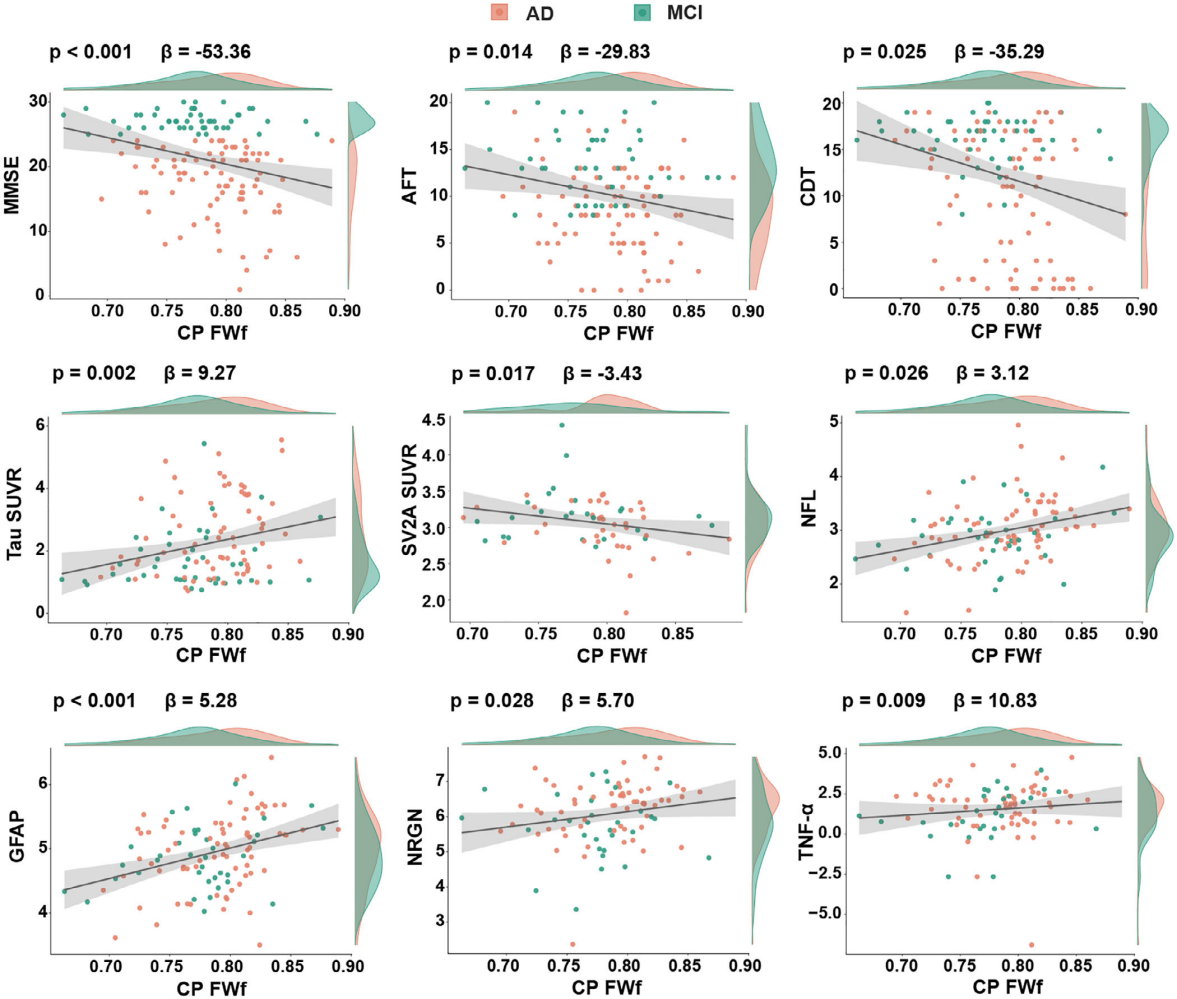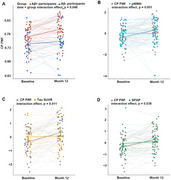# Choroid plexus free‐water reflects glymphatic dysfunction and neuroinflammation: a potential imaging marker in Alzheimer's disease

**DOI:** 10.1002/alz70856_097697

**Published:** 2025-12-24

**Authors:** Xinyuan Yang, Xiaomeng Xu, Junfang Zhang, Binyin Li

**Affiliations:** ^1^ Ruijin Hospital affiliated with Shanghai Jiao Tong University School of Medicine, Shanghai, Shanghai, China; ^2^ Ruijin Hospital affiliated to Shanghai Jiaotong University School of Medicine, Shanghai, Shanghai, China

## Abstract

**Background:**

Free‐water imaging of the choroid plexus (CP) is an index revealing components of the CP, and shows promise for early diagnosis and accurate monitoring of Alzheimer disease (AD).

**Methods:**

Our study evaluated free water fraction (FWf) of CP in 216 participants (133 Aβ+ participants and 83 Aβ‐ controls) continuously enrolled in the Ruijin NeuroBank of Alzheimer's Disease and Dementia (RJNB‐D) cohort. The ADNI dataset was used for external validation.

**Results:**

Following adjustment for age, sex, and ApoE genotype, the CP FWf and decreased diffusion tensor image analysis of the perivascular space (DTI‐ALPS) index were independently associated with Aβ positivity in both RJNB‐D and ADNI datasets (all *p* < 0.05, Figure 1A and B). In Aβ+ participants, DTI‐ALPS mediated the association between CP FWf and periventricular white matter hyperintensity (pWMH) (mediated effect = 35.76%). CP FWf was associated with cortical Tau accumulation, synaptic loss, hippocampal and cortical atrophy, impaired cognitive performance, and elevated levels of NFL, GFAP, NRGN, and TNF‐α. (all *p* < 0.05, Figure 2). Longitudinally, CP FWf increased faster in Aβ+ participants than Aβ‐ controls (time × group interaction effect *p* =  0.046, Figure 3A). The growth of CP FWf was associated with a reduction in DTI‐ALPS (*ρ* = ‐0.42, *p* = 0.006), and the growth rate of CP FWf surpassed that of pWMH, Tau, and GFAP. (Figure 3B, C and D).

**Conclusion:**

Elevated CP FWf indicates impaired glymphatic function and AD neurodegeneration, and can be a sensitive biomarker for AD progression. The study was registered on ClinicalTrials.gov (NCT05623124).